# Achievements and challenges in psychiatric education and training in Sri Lanka

**DOI:** 10.1192/bji.2021.35

**Published:** 2023-02

**Authors:** Aruni Hapangama, K.A.L.A. Kuruppuarachchi, Raveen Hanwella

**Affiliations:** 1 FRANZCP, Senior Lecturer, Department of Psychiatry, Faculty of Medicine, University of Kelaniya, Sri Lanka. Email: ahapangama@kln.ac.lk; 2 FRCPsych, Cadre Chair and Senior Professor of Psychiatry, Department of Psychiatry, Faculty of Medicine, University of Kelaniya, Sri Lanka; 3 FRCPsych, Cadre Chair and Senior Professor of Psychiatry, Department of Psychiatry, Faculty of Medicine, University of Colombo, Sri Lanka

**Keywords:** Psychiatry, undergraduate curriculum, internship, challenges, South Asia

## Abstract

When compared with other Asian countries, psychiatric education and training in Sri Lanka has made significant developments during the past two decades, such as introducing psychiatry as a separate final year subject in the undergraduate medical curricula. However, further developments in psychiatric training in medical education are needed.

Sri Lanka recorded a disability-adjusted life-year rate (per 100 000 population) of 2800.82 in 2017,^1^ but one of the biggest challenges in meeting the needs of people with mental illnesses has been the severe shortage of trained human resources: for example, the country has only 0.52 consultant psychiatrists per 100 000 of the population.^2,3^

## Undergraduate psychiatry education

Before the year 2000, undergraduate psychiatric teaching in Sri Lanka was limited to lectures and clinical attachments in the third and/or fourth years and a few tutorials in the final year. What was taught in those 3 years was assessed only at the final MBBS examination and limited to one structured essay question and a couple of multiple-choice questions in the medicine paper. However, with the landmark efforts of the University of Colombo, psychiatry was introduced as a final year subject with a separate paper. The other state universities followed, and some universities also introduced basic psychology lectures during the first 2 years of the medical curriculum.

Another significant achievement was the approval of the co-curriculum in psychiatry by the University Grants Commission of Sri Lanka. This is now adopted by all state universities, unifying teaching and assessments across the universities.

Since 2018, psychiatry has been a final year subject considered for the merit list for internship with the other four major clinical specialties: clinical medicine, surgery, obstetrics and gynaecology, and paediatrics. This has been a crucial and important step that would motivate students to study psychiatry. In addition, shifting of the core clinical teaching in psychiatry to the final year in Sri Lankan state universities gives students the opportunity of concurrent learning of psychiatry with the other four disciplines. It also emphasises the relationship between the five specialties and enables students to understand better the liaison model of psychiatry and the importance of mental health issues in all clinical specialties. Increasing the clinical training in psychiatry during the undergraduate years will improve students’ clinical competency in diagnosing mental illnesses and helps development of core soft skills such as listening, empathy, effective communication and humanitarian values.^4^ A study done among students of a state medical school showed that the knowledge and attitudes of the medical students towards psychiatry changed after introduction of the new curricula and the expansion of the undergraduate training programme in psychiatry.^5^

## Postgraduate education in psychiatry

A medical officer who passes the selection examination enters the MD training programme in psychiatry conducted by the Postgraduate Institute of Medicine, University of Colombo. The MD programme in psychiatry runs for 3 years and those who get through their MD examination can train in an approved centre overseas, as part of the medical training initiative (MTI) programme of the UK or a hospital-sponsored placement in Australia.

In the past, retention of psychiatric trainees in Sri Lanka had been a significant problem, as the country lost most of its psychiatry trainees, who for many personal reasons did not return from their overseas training.^6^

However, reports show that, in addition to increasing numbers of candidates sitting the selection examination for a MD in psychiatry, there is an encouraging trend with most trainees who had gone for overseas training to return home.^7^

### Postgraduate diploma in psychiatry

In 2007, to mitigate the treatment gap due to the lack of qualified specialists, the Postgraduate Institute of Medicine at the University of Colombo introduced a new psychiatry programme. This programme comprises a 1-year training course, leading to a diploma in psychiatry. Diploma holders serve mostly in out-patient clinics under the supervision of a consultant psychiatrist.

### Continuing professional development opportunities for board-certified specialists

Currently, besides self-guided learning, the Sri Lanka College of Psychiatrists provides an informal continuing professional development programme.

### Training of specialists in other disciplines

Board-certified specialists in psychiatry are invited to provide teaching and learning activities for postgraduate trainees in medicine and family medicine. Trainees in medicine and family medicine also have to undergo short appointments in clinical psychiatry.

## Areas that need improvement

### Internship in psychiatry

The main aim of a medical curriculum is to train a person to effectively manage a patient at the level of a family practitioner and give a grounding in the main clinical specialties that will be retained even after doctors with the basic medical degree specialise in their fields of interest. The internship is the key to the transition from being a medical undergraduate to a practising doctor.

Currently in Sri Lanka, graduating doctors can choose two specialties from the four traditional main clinical specialties and train as an intern for 6 months in each of the two selected. Psychiatry is not an option yet. This is neither logical nor prudent. Both Australia and the UK have moved away from the traditional apprenticeship model of internship and have structured programmes with clear learning objectives.^8,9^

The Medical Board of Australia, which sets a broad structure for internship training in Australia, requires a total duration of 47 weeks of full-time or equivalent training.^8^ Interns need to complete at least 10 weeks each in medicine and surgery, and at least 8 weeks in emergency medical care. They can spend the rest of the time in a range of specialties, including psychiatry and general practice.

In the UK too, Foundation Year 1 has replaced the traditional internship year.^9^ The trainee doctor is expected to complete 20 ‘foundation professional capabilities’, including knowledge, skills and behaviours, and to meet a minimum standard. The propotion of psychiatry has gradually being increased as per the 2016 foundation programme curriculum.^9^

A World Health Organization policy paper estimates that by 2030, the leading global disease burden will be untreated mental health problems (13%), particularly depression, and mental illness will be the principal cause of mortality and morbidity globally.^10^ In addition, psychiatrists or doctors with some mental health expertise will be expected to manage the behavioural and psychiatric problems of patients with dementia, self-harm and substance use problems. It will be crucial that all doctors in Sri Lanka have a minimal level of competence in managing these issues. Although having psychiatry as a final year merit subject is a major step, this is not sufficient. To ensure that a doctor gets practical experience in managing these common psychiatric and mental health problems, a period of practical training after medical school would be vital. An internship will be a valuable option to ensure these objectives. We propose an internship in psychiatry of 6 months added to the current mandatory internship period of one year. The intern can choose between psychiatry or other clinical specialties such as ear, nose and throat (ENT), ophthalmology, dermatology, emergency medicine, family medicine or community-based disciplines during this period. We postulate that those who are already interested in psychiatry will take up psychiatry and the internship will consolidate the skills gained during their final year. In addition, making this particular rotation elective would also minimise problems in finding an adequate number of supervisors and units where they can be placed, as some hospitals currently do not have adequate infrastructure.

A principal objection to the proposal would be the lengthening of the period of internship. However, this might be overcome to some extent by incentives such as naming the extended period a senior internship, with a corresponding increase in salary.

We also propose a more objective and structured assessment of a doctor's performance in internship than a signature by the consultant.

Despite a heavy burden of psychiatric morbidity, the only South Asian country to offer internship in psychiatry is India, where medical students complete a 2-week mandatory psychiatry internship.^11^ If Sri Lanka is to take up this pioneering role and assess its feasibility and success, other low- and middle-income countries may follow similar or modified models depending on their resources. Psychiatrists have to play a large role in convincing medical regulatory bodies and other stakeholders of the need for this proposed model.

### Training in research methodology

Another area that needs urgent attention is encouraging and training in research methodology related to psychiatry.^12^ There is a significant lack of funds for research in mental health in Sri Lanka and it is time that authorities allocate sufficient funds to increase the research capacity.

## Conclusions

Although the Sri Lankan psychiatric fraternity can be proud of their achievements in terms of educational and training needs, we need to be mindful that psychiatry is no longer an isolated discipline, and proper education and training of future medical practitioners will have widespread and critical health and economic implications.
